# *In Vitro* Chondrogenesis Transformation Study of Mouse Dental Pulp Stem Cells

**DOI:** 10.1100/2012/827149

**Published:** 2012-08-02

**Authors:** Shahrul Hisham Zainal Ariffin, Shabnam Kermani, Rohaya Megat Abdul Wahab, Sahidan Senafi, Zaidah Zainal Ariffin, Mohamad Abdul Razak

**Affiliations:** ^1^School of Biosciences and Biotechnology, Faculty of Science and Technology, Universiti Kebangsaan Malaysia, 43600 Bangi, Selangor, Malaysia; ^2^Department of Orthodontic, Faculty of Dentistry, Universiti Kebangsaan Malaysia, Jalan Raja Muda Abdul Aziz Road, 50300 Kuala Lumpur, Malaysia; ^3^Department of Microbiology, Faculty of Applied Science, Universiti Teknologi MARA, 40450 Shah Alam, Malaysia; ^4^College of Medical Sciences, Allianze University, Waziria Medical Square, Jalan Bertam 2, 13200 Kepala Batas, Malaysia

## Abstract

A major challenge in the application of mesenchymal stem cells in cartilage reconstruction is that whether the cells are able to differentiate into fully mature chondrocytes before grafting. The aim of this study was to isolate mouse dental pulp stem cells (DPSC) and differentiate them into chondrocytes. For this investigation, morphological, molecular, and biochemical analyses for differentiated cells were used. To induce the chondrocyte differentiation, DPSC were cultured in chondrogenic medium (Zen-Bio, Inc.). Based on morphological analyses using toluidine blue staining, proteoglycan products appear in DPSC after 21 days of chondrocyte induction. Biochemical analyses in differentiated group showed that alkaline phosphatase activity was significantly increased at day 14 as compared to control (*P* < 0.05). Cell viability analyses during the differentiation to chondrocytes also showed that these cells were viable during differentiation. However, after the 14th day of differentiation, there was a significant decrease (*P* < 0.05) in the viability proportion among differentiated cells as compared to the control cells. In RT-PCR molecular analyses, mouse DPSC expressed *Cd146* and *Cd166* which indicated that these cells belong to mesenchymal stem cells. *Coll I* and *Coll II* markers showed high expression after 14 and 21 days, respectively. In conclusion, this study showed that DPSC successfully differentiated into chondrocytes.

## 1. Introduction

In recent years, new cellular therapy methods have opened a clear horizon for treatment of various injuries and diseases. Globally, many people experience at least one form of cartilage tissue injuries. These injuries are usually the result of traumas or tissue degeneration due to age related diseases. Since there is lack of vascular system in cartilage tissue or an active supply system for cartilage tissues that covering at the ends of bones, growth factors that exist in blood that is needed for cellular healing are thus limited. This results in a restricted ability of self-healing in these tissues [1]. 

The demand for chondrocyte grafting has increased in recent years, but there are limitations as it cannot be used for vast cartilage injuries. Access to other cellular resources for the production of cartilage tissues and grafting is thus crucial in the treatment of cartilage injuries [2]. Stem cells can be an alternative resource for the purpose of cartilage injury healing. The main focus for regenerative research is to explore the potential resource for cellular therapy. Mesenchymal stem cells can be found in the bone marrow [3], muscle tissues [4], cartilages [5], tendons [6], umbilical cord [7], and umbilical cord blood [8]. Among stem cells with mesenchymal origin, those from the bone marrow have received more attention [9]. A bone marrow stromal cell is a type of mesenchymal stem cell found in the bone marrow. They are able to give rise to bones and cartilages [10]. One point that should be noted in the definition of these cells is that their attachment to the bottom of culture dishes could be used as a way of recognizing them [11]. Cells which attach to the bottom of culture dishes are heterogenic, consisting of progenitor and stem cells [12] and their fate changes depending on the differentiation environment. For example, ascorbic acid, nonorganic phosphates and dexamethasone can influence the differentiation of these cells into osteoblasts [13], while transforming growth factor B can impose chondrogenic markers in these cells [14].

Teeth are rich, unique and accessible sources of mesenchymal stem cells that are suitable for applied research and tissue engineering applications [15]. Following ectomesenchymal interactions (confrontation) occurring between the dental ridges and its underlying mesenchyme, dental layers are formed. Dental layers are further differentiated into dental organs; dental papilla and dental follicles. Finally, major dental structures and periodontal tissues are developed. Meanwhile, dental pulp is formed from mesenchymal neural crests with multipotent abilities [16]. The purpose of this study was to investigate the chondrogenic capacity of mouse dental pulp cells and identify DPSC differentiation into chondrocytes through morphological, molecular, and biochemical analyses. 

## 2. Materials and Methods

### 2.1. Isolation of DPSC

In this experimental study, teeth were obtained from mouse aged 6–8 weeks. Surfaces of teeth were cleaned with phosphate-buffered saline (PBS) and kept in sterile PBS solution containing 1% (v/v) penicillin-streptomycin at 4°C. The teeth were then extracted from jaw using a sterile scalpel, while dental pulps were removed from teeth using sterile forceps. After this, the crown and root were cut with surgical scissors. The dental pulp was washed with PBS containing 1% (v/v) penicillin-streptomycin and was placed in 4 U of collagenase type 1 at 37°C. Single cells from dental tissues were obtained by pipetting the cells several times in *α*-modified Eagle's medium (AMEM). After this, the cells were centrifuged at 1200 g for 10 minutes. The pellet was resuspended with complete medium (AMEM supplemented with 20% v/v fetal bovine serum) and cultured in a 6-well plate. For cultivation, cells were transferred into T25 flasks containing complete medium and kept in incubator with a temperature of 37°C and humidity of 95% and 5% CO_2_.

### 2.2. Differentiation to Chondrocyte Cells

Approximately 1 × 10^5^ cells/mL were transferred to 24-well plates and kept in incubator with a temperature of 37°C and humidity of 95% and 5% CO_2_. After washing the cells with 1 X PBS, the cells were placed in chondrogenic medium (Zen-Bio, Inc.) for 21 days to observe for chondrocyte differentiation. Spent medium was replaced with fresh complete medium after every 3 days.

### 2.3. Chondrocyte Cell Staining

After 21 days of culturing in chondrogenic medium, the cells were first fixed in formaldehyde 4% (v/v) for 2 hours. Then, they were treated sequentially with alcohol 75% (v/v), 95% (v/v), and 100% (v/v). Finally, the cells were stained by toluidine blue for 2 minutes at room temperature. 

### 2.4. RNA Isolation for Chondrocyte Cells

Total RNA was extracted from these cells using Trizol at day 1, 14 and 21. The cells were detached from the T25 flask using 0.25% (v/v) trypsin/EDTA. Cells were then centrifuged at 1200 g for 10 minutes, and the obtained pellet was lysed with 1 mL TRIZOL (Regent-Total RNA Isolation Regent) (Invitrogen, USA) for 5 minutes at room temperature. Approximately 0.2 mL chloroform (SYSTERM) was added into the sample and vortexed for 15 seconds. This was followed by centrifugation at 12000 g for 15 minutes at 4°C. The colorless aqueous phase was taken as the RNA. This phase was transferred into new tube, and 0.5 mL of 100% (v/v) isopropanol was added and incubated at room temperature for 10 minutes. After that, the sample was centrifuged at 12000 g for 10 minutes at 4°C. The supernatant was discarded, and the RNA pellet was washed with 15% (v/v) ethanol. The sample was centrifuged again at 7500 g for 5 minutes at 4°C. The supernatant was discarded, and the RNA pellet dried at room temperature for 5 minutes before resuspending in 25 mL free nuclease water and incubated at 55°C for 15 minutes. The obtained RNA was stored at -80°C until use.

### 2.5. Reverse Transcriptase Polymerase Chain Reaction (RT-PCR)

Materials for RT-PCR ( Access Quick RT-PCR Kit System, Promega) were 5X reaction buffer AMV/Tfi, dNTP mix (10 mM), forward and reverse primers (50 pmol), MgSO4 (25 mM), reverse transcriptase (5 U/*μ*L), template RNA (0.5 *μ*g), and nuclease free water. Briefly, RT-PCR reaction was conducted using chondrocyte cells primers, that is, including *Coll  I* and *Coll  II* (Table 1). RT-PCR program was conducted with thermocycler conditions as follows: primary cDNA synthesis at 45°C for 45 minutes and deactivation of the AMV reverse transcriptase at 94°C for 2 minutes. Secondary cDNA synthesis, including 40 cycles of denaturation, at 94°C for 30 seconds, with 1 minute annealing using primer-specific temperature and 2 minutes of primary extension at 68°C were conducted. Final extensions at 68°C for 7 minutes were performed. Two percent agarose gel (Vivantis Inc. USA) and 1X buffer TAE was used for gel electrophoresis. After electrophoresis, gel was stained with EtBr and observed under UV light.

### 2.6. Alkaline Phosphatase Activity

Alkaline phosphatase (ALP) activity was assayed enzymologically. The DPSC was seeded at a density of 1 × 10^3^ cells/mL in 96-well plates. At day 1, 5, 7, 10, 14, and 21 of culture with differentiation medium and control medium, the ALP activity of DPSC was determined using an ALP assay. Spent control and differentiation mediums were replaced with fresh medium after every two days. After washing with 1X PBS, the cells were incubated in 0.1 M NaNO_3_-Na_2_CO_3_ buffer (pH 10.0) (w/v) (R&M, U.K) containing 1% ( v/v) Triton X100 (Sigma, USA) and 2 mM MgSO_4_ (w/v) (Sigma, USA). Subsequently, 6 mM P-Nitrophenyl Phosphate (w/v) (Sigma, USA) was added as substrate to each 96-well and incubated for 30 minute at 37°C. Finally, 1.5 M NaOH (sodium hydroxide) (w/v) (Labguard, USA) was added to stop the enzyme substrate reaction. Optical density (OD) readings were taken at wavelength of 405 nm using a spectrophotometer. 

### 2.7. MTT Assay for Chondrocyte Cells

Approximately 1 × 10^3^ cells/mL was placed in 96-well dishes after incubation at 37°C for 24 hours. The cells were washed with 1X PBS and divided into two groups, that is, control and chondrocyte induction. Following this, the cells were cultured in the chondrocyte medium (induction group) and complete medium (control group) for 1, 3, 5, 7, 10, 12, 14, 16, 18, and 21 days. The sample design included three replicates for each treatment and five absorbance measurements for each sample. Twenty microlitre of MTT (5 mg/mL in phosphate buffered saline, PBS) were added to each well and the samples incubated at 37°C for 4 hours. After removing the mixture, the insoluble purple-blue formazan crystals were dissolved with 200 *μ*L DMSO (dimethyl sulfoxide). The cells were then incubated for 15minutes at room temperature. The absorbance was thereafter measured at 570 nm using an ELISA reader. 

### 2.8. Statistical Analysis

Statistical comparison between the chondrocyte differentiated groups and control were carried out using *t*-test. Observed differences were considered statistically significant when  *P* < 0.05. 

## 3. Results

**Figure 1:**
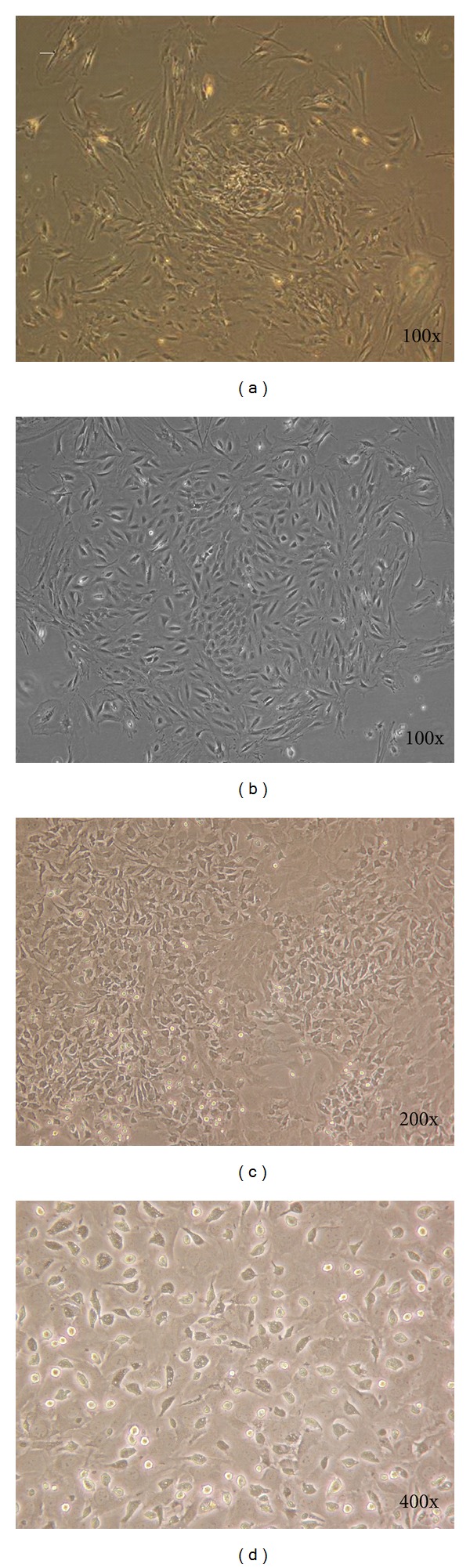
Characteristics of isolated and *in vitro* mouse DPSC colony formation after cultured DPSC at the first passage (a and b). Cell after 48 hours after culture (c) and fibroblastic-like cells shape (d).

In this study, after dental pulp tissues were digested with collagenase enzyme, spherical cells appeared. After 24 hours, cells which were placed in culture plate changed into fibroblastic shape with stretched form and circular nucleus. The morphology of mouse dental pulp cells and chondrogenic ability of these cells is illustrated in Figures 1(a) and 1(b). To differentiate DPSC to chondrocytes, only the fourth cell passage was used. After 21 days of culture in chondrogenic induction medium, the cellular morphological characters had changed.

**Figure 2:**
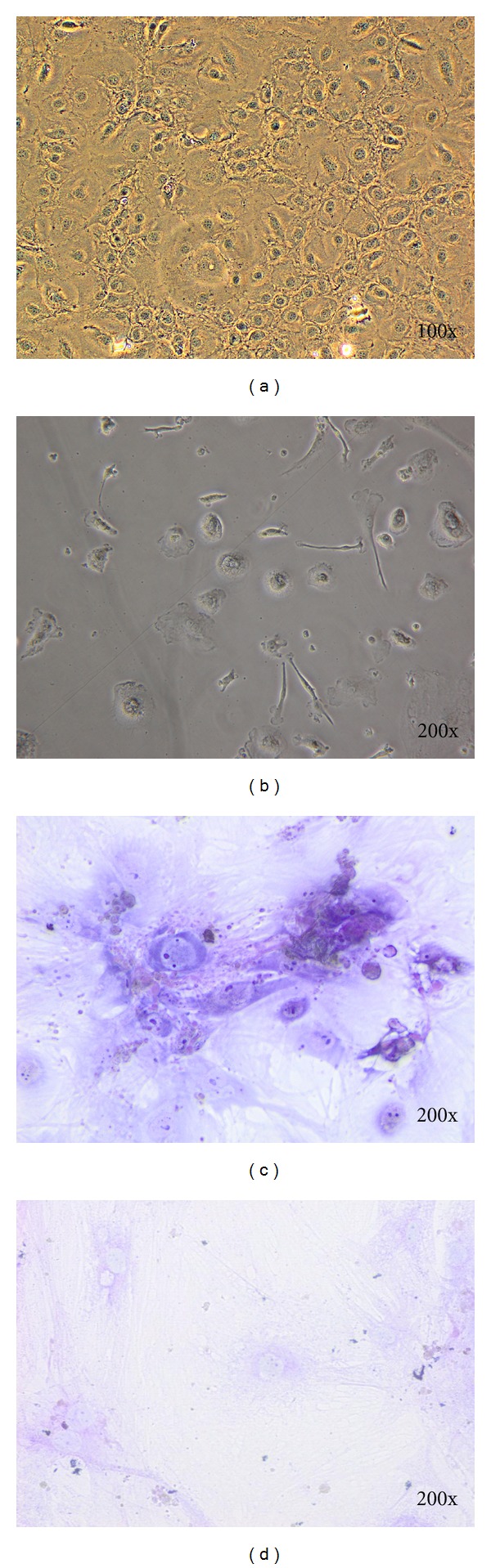
Characteristics of chondrocyte derived from mouse DPSC. After chondrogenic induction, the cytoplasm contracted toward the nucleus and formed spherical cells without branch (a and b). Glycosaminoglycans in chondrocyte tissue were stained by toluidine blue and showed blue in colors (c). Control groups without chondrogenic induction (d).

After chondrogenic induction, the cytoplasm contracted toward the nucleus and formed round shape cells without branches (Figures 2(a) and 2(b)). Cells that have been cultured in chondrocyte differentiation medium were stained by toluidine blue to produce blue colors (Figure 2(c)). Toluidine blue staining revealed an increased production of glycosaminoglycan during induction, a phenomenon noticed only among chondrocyte cells.

**Figure 3:**
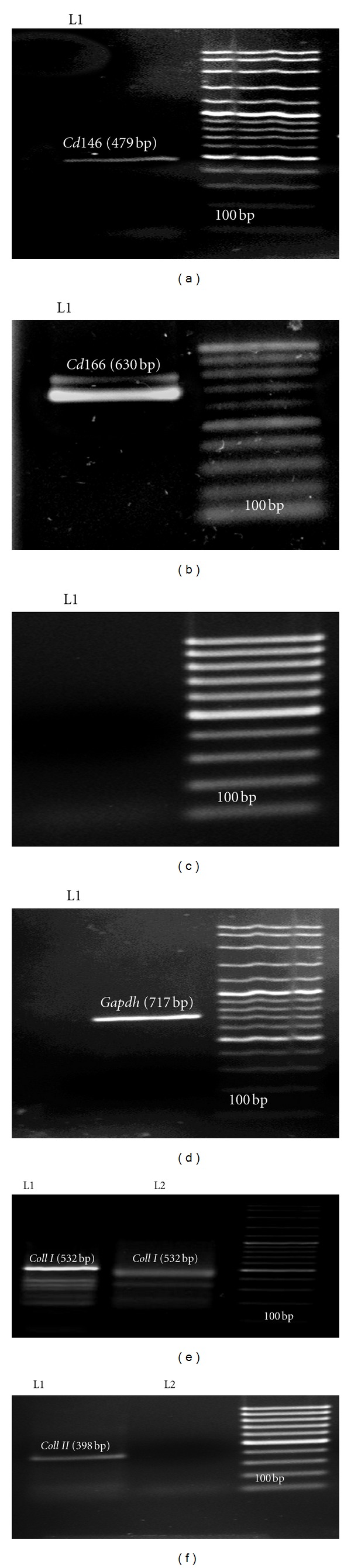
RT-PCR analyses of mouse DPSC in 1% (w/v) Agarose. (a) *Cd146 *(479 bp), (b) *Cd166* (630 bp), (c) *Cd 31*(355 bp), (d) *Gapdh* as a house keeping gene (717 bp). (e, L1) The *Coll  I* marker (532 bp) was after 14 days induction and (e, L2) before induction. (f, L1) *Coll  II* after 21 days induction and (f, L2) before induction.

In RT-PCR, mouse DPSC where showed to express *Cd1*46 and *Cd1*66 indicating that these cells belong to mesenchymal stem cells (Figures 3(a) and 3(b)). Since these cells did not express *Cd*31 (Figure 3(c)), it showed that they do not belong to hematopoietic stem cells. On the other hand, *Coll  I* marker was shown to be highly expressed after 14 days of induction (Figure 3(e), L1) as compared to before induction (Figure 3(e), L2) whereas activation of *Coll  II* as mature chondrocyte cells markers was observed after 21 days treatment with chondrocyte induction medium (Figure 3(f), L1). On the other hand, before induction (Figure 3(f), L2) showed no amplification of *Coll  II*.

**Figure 4:**
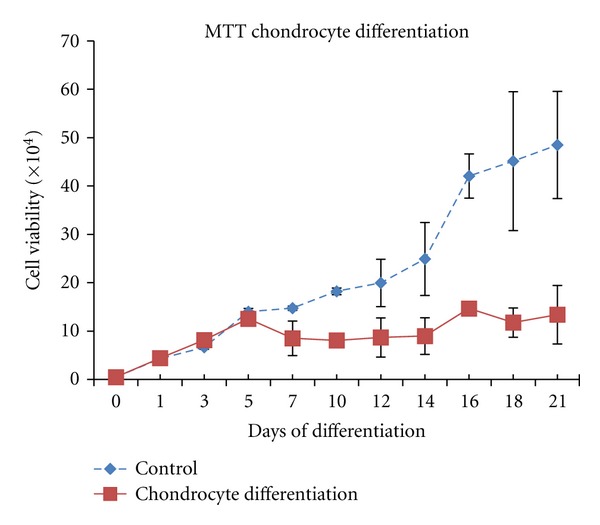
Cell viability by using MTT assay. The results were presented as mean ± SD. Statistical analysis was conducted by *t*-test. Comparison of data between controls and groups showed significant difference after 14 days onwards (*P* < 0.05; *n* = 3).

**Figure 5:**
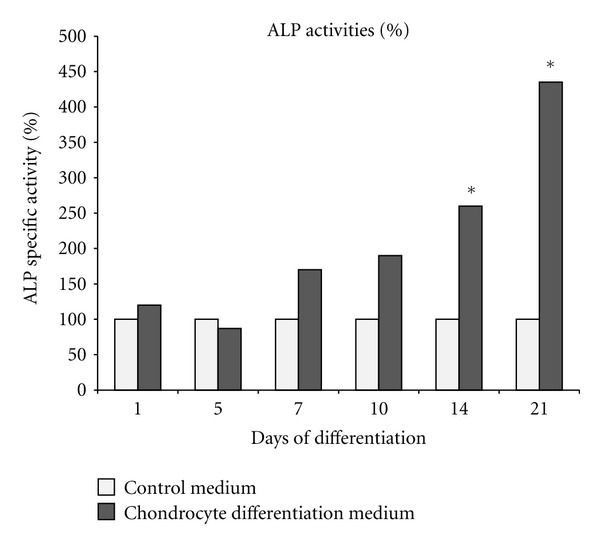
Alkaline Phosphatase (ALP) assay. Statistical analysis using paired *t*-test. Comparison of data between controls and differentiated groups showed significant difference (*) after 14 days and 21 days (*P* < 0.05; *n* = 3).

Cell viability analyses using MTT assay during differentiation stage showed that the proliferation ability of differentiated cells is weaker as compared to the control. Statistical analyses also demonstrated significant differences (*P* < 0.05) between the control and chondrogenic induction group at day 7 until 21 (Figure 4). ALP activity of DPSC cultured in chondrogenic differential medium was detected using an ALP enzymological assay. After 14 days of culturing DPSC in the chondrogenic induced medium, results showed that most of the cells became alkaline phosphatase positive as compared to control cells which were cultured in AMEM and 15% (v/v) FBS. Enzymatic activity of differentiated cells reached the highest valueafter21 days (Figure 5).

## 4. Discussions

Expression of *STRO 1* and *CD146* as human markers are amongst DPSC characteristics. These two markers are expressed at the peripheral vascular and neural areas in dental pulp [17]. In our study, *Cd146* and *Cd166* markers were also shown to be expressed in the basal culture medium. Furthermore, Laino et al. [18] demonstrated that DPSC frozen for 2 years manifested not only the characteristics of fresh DPSC but also maintained the ability to differentiate into bone cells when cultured in appropriate medium [18, 19].

Despite the physical strength of cartilages, these cells are capable of self-repair after significant trauma or disease. Regenerative cartilages are thus important candidates for regenerative medicine [20]. Production of these cells is essential since cartilages have low cellular density. On the other hand, high numbers of cells are usually needed for cellular therapy [21]. Currently, various treatment methods are being used to reconstruct cartilages. One of the treatment approaches is cellular therapy using autologouschondrocytes [21]. Since chondrocytes show morphological changes and lose differentiation capability during *in vitro* culture, it is essential to find alternative cells that could retain this ability [22, 23]. 

Imabayashi et al. [24] showed that differentiated chondrocytes need three-dimensional culture to acquire differentiation characteristic. Application of mesenchymal cells in cartilage reconstruction was applied because it was believed that mesenchymal cells could differentiate into complete mature chondrocytes before grafting [24]. This approach guarantees chondrocytes transition to the targeted location and prevented unexpected differentiation in joint cartilage injury [25]. Hence, in this study we used *in vitro* methods to investigate the potency of DPSC to differentiate into chondrocytes. 

There are limitations in differentiation of mesenchymal stem cell into chondrocytes. However, growth factors like TGF and the absence of serum can be used in bone marrow mesenchymal stem cells to overcome these limitations [26]. Zuk et al. [27] used growth factor TGF-*β*1 during chondrocyte differentiation of mesenchymal stem cells originated from human adipose tissues. Sekiya et al. [28] reported that production of proteoglycans increased in stem cells derived from bone marrow by adding BMP-6 into culture medium. On the other hand, some studies revealed that ascorbic acid could stimulate *in vitro* differentiation and proliferation of various mesenchyme-derived cell types such as osteoblasts [29–31], adipocyte [32], and chondrocytes [33, 34]. 

The most appropriate factors needed to differentiate stem cells into chondrocytes have not been identified yet. However, the groups of TGF growth factors are important among chondrogenic inducing factors. These factors play a key role in regulation of cellular growth, cellular differentiation, cartilage, and bone development [35]. Some studies have shown that TGF isoforms are not merely enough for mesenchymal stem cell differentiation, and that additional factors such as BMP are needed to induce increment of proteoglycans [28]. DPSC under the influence of TGF with high cellular intensity that is present in Zen Bio medium were therefore used in this study as chondrogenic differentiation inducer.

As indicated by other studies, existence of *Coll  II* and proteoglycan are indicators of cartilage tissue [27, 36]. In this study, determination of *Coll  II* chondrocyte markers after differentiation revealed that these markers were expressed after 21 days in differentiated medium (Figure 3(f), L1). While *Coll  I* was observable before differentiation, it increased its expression after 14 days (Figure 3(e), L1 and 3(e), L2). Investigation of alkaline phosphatase activity was a proof for this claim as statistical analysis showed significant differences (*P* < 0.05) in differentiation among cells treated by chondrogenic medium as compared to control on the 14th and 21st day (Figure 5).

Cell viability during differentiation into chondrocytes (Figure 4) also showed that cells preserved their viability during differentiation, but after the 14th day of culture the cell viability ratio between differentiated and control cells was significantly decreased (*P* < 0.05). This shows that these cells lose their ability to proliferate during the differentiation process. 

In conclusion, this study indicated that DPSC with high proliferation rate had chondrogenic differentiation capacity. This type of stem cells might be suitable for autologous chondrocyte repair. 

## Figures and Tables

**Table 1:** Primes and the reaction conditions of RT-PCR.

Name	Sense primer	Antisense primer	Accession No.	Prod. length (bp)	Ann. *T* (^°^C)
*CD 146*	5^′^GGACCTTGAGTTTGAGTGG3^′^	5^′^CAGTGGTTTGGCTGGAGT3^′^	NM_023061	479	60
*CD 166*	5^′^AACATGGCGGCTTCAACG3^′^	5^′^GACGACACCAGCAACGAG3^′^	NM_009655	630	61
*CD31*	5^′^GGTCTT GTCGCAGTATCAG3^′^	5^′^ATGGCAATTATCCGCTCT3^′^	NM_001032378.1	355	58
*Gapdh*	5^′^CAACGGCACAGTCAAGG3^′^	5^′^AAGGTGGAAGAGTGGGAG3^′^	NM_008084	717	62
*Coll II*	5^′^TGGTGGAGCAGCAAGAG3^'^	5^′^ATGGGTGCGATGTCAAT3^'^	NM_001113515	398	53
*Coll I*	5^′^GAGTGCTGTGCTTTCTGC3^′^	5^′^CTCGGTGTCCCTTCATTC3^′^	NM_001113515	532	62.8

## References

[1] Hardingham TE, Maddison PJ, Isenberg DA, Woo P, Glass DN (1998). Articular cartilage. *Oxford Textbook of Rheumatology*.

[2] Esfandiary E, Amirpour N, Fesharaki M (2008). Access to chondrocyte culture, with alginate, in Iran. *Yakhteh*.

[3] Nakamizo A, Marini F, Amano T (2005). Human bone marrow-derived mesenchymal stem cells in the treatment of gliomas. *Cancer Research*.

[4] Jankowski RJ, Deasy BM, Huard J (2002). Muscle-derived stem cells. *Gene Therapy*.

[5] Eslaminejad MRB, Taghiyar L (2007). Mesenchymal stem cell purification from the articular cartilage cell culture. *Iranian Journal of Basic Medical Sciences*.

[6] Rui YF, Lui PPY, Li G, Fu SC, Lee YW, Chan KM (2010). Isolation and characterization of multipotent rat tendon-derived stem cells. *Tissue Engineering—Part A*.

[7] Covas DT, Siufi JLC, Silva ARL, Orellana MD (2003). Isolation and culture of umbilical vein mesenchymal stem cells. *Brazilian Journal of Medical and Biological Research*.

[8] Flynn A, Barry F, O’Brien T (2007). UC blood-derived mesenchymal stromal cells: an overview. *Cytotherapy*.

[9] Pittenger MF, Mackay AM, Beck SC (1999). Multilineage potential of adult human mesenchymal stem cells. *Science*.

[10] Johnstone B, Hering TM, Caplan AI, Goldberg VM, Yoo JU (1998). In vitro chondrogenesis of bone marrow-derived mesenchymal progenitor cells. *Experimental Cell Research*.

[11] Bauer S, Park J, Mark KVD, Schmuki P (2008). Improved attachment of mesenchymal stem cells on super-hydrophobic TiO_2_ nanotubes. *Acta Biomaterialia*.

[12] Ogura N, Kawada M, Chang WJ (2004). Differentiation of the human mesenchymal stem cells derived from bone marrow and enhancement of cell attachment by fibronectin. *Journal of oral science*.

[13] Choi KM, Seo YK, Yoon HH (2008). Effect of ascorbic acid on bone marrow-derived mesenchymal stem cell proliferation and differentiation. *Journal of Bioscience and Bioengineering*.

[14] Tuli R, Tuli S, Nandi S (2003). Transforming growth factor-beta-mediated chondrogenesis of human mesenchymal progenitor cells involves N-cadherin and mitogen-activated protein kinase and Wnt signaling cross-talk. *The Journal of Biological Chemistry*.

[15] Casagrande L, Cordeiro MM, Nör SA, Nör JE (2011). Dental pulp stem cells in regenerative dentistry. *Odontology*.

[16] Bluteau G, Luder HU, De Bari C, Mitsiadis TA (2008). Stem cells for tooth engineering. *European Cells and Materials*.

[17] Shi S, Gronthos S (2003). Perivascular niche of postnatal mesenchymal stem cells in human bone marrow and dental pulp. *Journal of Bone and Mineral Research*.

[18] Laino G, Carinci F, Graziano A (2006). In vitro bone production using stem cells derived from human dental pulp. *Journal of Craniofacial Surgery*.

[19] Gronthos S, Brahim J, Li W (2002). Stem cell properties of human dental pulp stem cells. *Journal of Dental Research*.

[20] Yang WD, Chen SJ, Mao TQ (2000). A study of injectable tissue-engineered autologous cartilage. *The Chinese Journal of Dental Research*.

[21] Brittberg M, Lindahl A, Nilsson A, Ohlsson C, Isaksson O, Peterson L (1994). Treatment of deep cartilage defects in the knee with autologous chondrocyte transplantation. *The New England Journal of Medicine*.

[22] Lindahl A, Brittberg M, Peterson L (2001). Health economics benefits following autologous chondrocyte transplantation for patients with focal chondral lesions of the knee. *Knee Surgery, Sports Traumatology, Arthroscopy*.

[23] Marijnissen WJCM, Van Osch GJVM, Aigner J, Verwoerd-Verhoef HL, Verhaar JAN (2000). Tissue-engineered cartilage using serially passaged articular chondrocytes. Chondrocytes in alginate, combined in vivo with a synthetic (E210) or biologic biodegradable carrier (DBM). *Biomaterials*.

[24] Imabayashi H, Mori T, Gojo S (2003). Redifferentiation of dedifferentiated chondrocytes and chondrogenesis of human bone marrow stromal cells via chondrosphere formation with expression profiling by large-scale cDNA analysis. *Experimental Cell Research*.

[25] Heng BC, Cao T, Eng HL (2004). Directing stem cell differentiation into the chondrogenic lineage in vitro. *Stem Cells*.

[26] Pittenger MF, Mackay AM, Beck SC, Murphy JM, Barry FP, Chichester CO (1998). Chondrogenic differentiation of cultured human mesenchymal stem cells from marrow. *Tissue Engineering*.

[27] Zuk PA, Zhu M, Ashjian P (2002). Human adipose tissue is a source of multipotent stem cells. *Molecular Biology of the Cell*.

[28] Sekiya I, Colter DC, Prockop DJ (2001). BMP-6 enhances chondrogenesis in a subpopulation of human marrow stromal cells. *Biochemical and Biophysical Research Communications*.

[29] Wang Y, Singh A, Xu P, Pindrus MA, Blasioli DJ, Kaplan DL (2006). Expansion and osteogenic differentiation of bone marrow-derived mesenchymal stem cells on a vitamin C functionalized polymer. *Biomaterials*.

[30] Takamizawa S, Maehata Y, Imai K, Senoo H, Sato S, Hata RI (2004). Effects of ascorbic acid and ascorbic acid 2-phosphate, a long-acting vitamin C derivative, on the proliferation and differentiation of human osteoblast-like cells. *Cell Biology International*.

[31] Tsuneto M, Yamazaki H, Yoshino M, Yamada T, Hayashi SI (2005). Ascorbic acid promotes osteoclastogenesis from embryonic stem cells. *Biochemical and Biophysical Research Communications*.

[32] Lin TM, Tsai JL, Lin SD, Lai CS, Chang CC (2005). Accelerated growth and prolonged lifespan of adipose tissue-derived human mesenchymal stem cells in a medium using reduced calcium and antioxidants. *Stem Cells and Development*.

[33] Sato H, Takahashi M, Ise H (2006). Collagen synthesis is required for ascorbic acid-enhanced differentiation of mouse embryonic stem cells into cardiomyocytes. *Biochemical and Biophysical Research Communications*.

[34] Ishikawa S, Iwasaki K, Komaki M, Ishikawa I (2004). Role of ascorbic acid in periodontal ligament cell differentiation. *Journal of Periodontology*.

[35] Wozney JM, Rosen V (1998). Bone morphogenetic protein and bone morphogenetic protein gene family in bone formation and repair. *Clinical Orthopaedics and Related Research*.

[36] Hashemibeni B, Razavi S, Esfandiary E, Karbasi S, Mardani M (2008). Induction of chondrogenic differentiation of human adipose derived stem cells with TGF-*β*3 in pellet culture system. *International Journal of Basic Medical Sciences*.

